# Editorial: Current Concept and Translational Study in ALS–FTD Spectrum: From Genetics, Neuroinflammation to Neurodegeneration

**DOI:** 10.3389/fnmol.2022.935115

**Published:** 2022-06-10

**Authors:** Wenting Guo, Zhang-Yu Zou, Yue Leng, Ding-Ding Shen, Sheng Chen

**Affiliations:** ^1^Laboratory of Neurobiology, VIB & KU Leuven Center for Brain & Disease Research, Leuven, Belgium; ^2^KU Leuven-Stem Cell Institute (SCIL), KU Leuven, Leuven, Belgium; ^3^Department of Neurology, Fujian Medical University Union Hospital, Fuzhou, China; ^4^Department of Psychiatry and Behavioral Sciences, University of California, San Francisco, San Francisco, CA, United States; ^5^Department of Neurology, Ruijin Hospital, Shanghai Jiao Tong University School of Medicine, Shanghai, China; ^6^Co-innovation Center of Neuroregeneration, Nantong University, Nantong, China

**Keywords:** ALS-FTD, neuroinflammation, neurodegeneration, genetics, translational studies, treatment

In this Research Topic, we present a comprehensive collection of research articles, data reports, and reviews contributed from leading experts in the ALS-FTD spectrum research field. This Research Topic highlights several key mechanisms in the pathogenesis of ALS-FTD, including neuroinflammation, DNA damage, and protein aggregations. A deep dissection of the underlining mechanisms will push forward the achievements of effective treatments.

## Understand the Foundation—Genetics

The past decades have witnessed a significant progression in identifying the genetic architecture of ALS-FTD spectrum disorder (van Es et al., 2017). Clear evidence has been found to support the fact that the ALS-FTD spectrum has a strong genetic influence and is highly heterogeneous in its genetic causes. To facilitate analysis of genes and variants related to ALS and other typical motor neuron disorders, Zhao et al. developed a one-stop database of motor neuron disease-related genetic information, called Gene4MND. This database collected most of the up-to-date genetic discoveries in all types of motor neuron disorders including ALS, primary lateral sclerosis (PLS), progressive muscular atrophy (PMA), and progressive bulbar palsy (PBP). Featured by a user uploading tool and functional analyzing function, Gene4MND provided a very efficient platform to update new genetic findings and to serve as a systematic analyzing platform based on comprehensive genetic evidence. An interesting study from Francisco Vázquez-Costa et al. reported that mutations in *Presenilin-1 (PSEN1)* can cause PLS-like syndrome. PLS is an upper motor neuron degenerative disorder that has been long debated whether it should be categorized as ALS. With the fact that PSEN1 has been identified as the most common cause of familial Alzheimer's disease (Pigino et al., 2003), this study further validated the close linkage between typical motor neurons disorders and dementias. It might be possible to eventually determine that different motor neuron disorder subtypes and different dementia sub-types share the same genetic makeup. Therefore,it will be of interest if a public database can be developed to cover both motor neuron disorders and dementias.

## Dive Into the Reason—Mechanisms

Multiple mechanisms have been suggested to contribute to the disease progression of the ALS-FTD spectrum based on the complexity of genetic architecture, physiological status, and environmental factors (Ferrari et al., 2011; Ahmad et al., 2016; Nguyen et al., 2018). Nevertheless, neuronal degeneration has been considered as the root cause and an immense amount of research has been dedicated to exploring common mechanisms of different subtypes of this disease spectrum. An original research article from Bottero et al. investigated gene expression networks by directly using spinal cord motor neurons from ALS patients and controls. This study adapted seven independent microarrays to find “switch genes” and related pathways. Remarkably, two transcriptional factors (*ELK1* and *GATA2*) that are involved in oncogenesis and immune response have been identified as the key master regulators to the “switch genes.” Accordingly, a series of pathways involved in cancer and cell cycle have been identified as the most enriched pathways in the ALS patient group in this study. Considering that the DNA damage is well recognized as a vital promoting factor in cancer as well as in neurodegeneration, it is not surprising that the cancer-related genes and pathways popped up from the microarrays. Closely related with this discovery, Wang et al. presented an in-depth review article focusing on the involvement of DNA damage and repair deficiency in the ALS-FTD spectrum. This review emphasized on the DNA repair defects induced by causative genes of the ALS-FTD spectrum, highlighted the consequences of DNA repair defects in disease progression, and proposed the therapeutic potential of targeting the DNA damage repair process to ameliorate neurodegeneration. In addition, a review article from Tejido et al. focused on an ALS causative gene *Fused In Sarcoma (FUS)* that functions as a DNA/RNA binding protein. This review highlighted the toxicity of FUS aggregations in nuclear and cytoplasm, discussed the involvement of the liquid-liquid phase separation (LLPS) phenomenon in forming FUS aggregation, and suggested rescuing the toxicity of FUS aggregation by modulating histone acetylation. Another FTD causative gene *progranulin (GRN)* has also been intensively discussed by a review article from Terryn et al. Interestingly, the biological function of the GRN protein is involved in regulating inflammation, tumor cell growth, and metastasis. This review provided a systematic overview of the multifaceted regulation of GRN and the corresponding therapeutic avenues by elevating GRN levels. Furthermore, neuroinflammation has also been highlighted by a focused review article from Liu et al. in our topic Research Topic. This review summarized the current research findings about neuroinflammation in ALS-FTD patients, new hypotheses based on animal studies, and the state of clinical trials targeting inflammation. The authors have thoroughly discussed both innate and adaptive immune responses and proposed that human induced pluripotent stem cell (iPSC) technology will serve as a more reliable tool to further explore neuroinflammation in the ALS-FTD spectrum. Aside from the molecular mechanisms in cellular settings, a comprehensive review from Kwong et al. summarized 40 years of cerebrospinal fluid (CSF) toxicity studies in the ALS-FTD spectrum. This review is so far the most complete summary of the early evidence of *in vitro* neurotoxicity of this disease spectrum and proposed a broad impact of the toxic CSF circulatory system to major disease mechanisms including neuroinflammation, glutamate excitotoxicity, proteotoxicity, and oxidative stress. Therefore, further research on both cellular and extra cellular toxicity are needed to get a clear picture of pathogenesis.

## Make a Difference—Treatments

The final goal of exploring disease mechanisms is to find effective treatments for the patients. Hundreds of agents have been proposed as candidates for ALS-FTD treatment. However, most clinical trials failed, which indicated the unsuccessful translation from bench to bedside. Two important reasons may contribute to this failure: inadequacy of ALS-FTD models and lack of presymptomatic biomarkers. For the first reason, the animal models we used were modified rodents carrying fALS genetic mutations, which cannot fully recapitulate the complete patho-physiological and phenotypic spectrum of ALS patients. For the second reason, the lack of presymptomatic biomarkers and the delay in clinical diagnosis have significantly limited the therapeutic potential of putative disease-modifying drugs.

In our Research Topic, multiple therapeutic approaches including gene therapy, HDAC inhibition, protein therapy, and cell therapy have been summarized and proposed to target the DNA damage repair system, protein aggregation formation, and neuroinflammation. A more comprehensive understanding of ALS pathogenesis as well as identifying presymptomatic biomarkers may improve the therapeutic effect of the ALS-FTD spectrum in the future (Xu et al., 2021). Taking together the extremely high genetic and clinical heterogeneity of this spectrum, we suggest that it will be of use to adapt cocktail treatments based on the pathological condition and the stages of disease progression of individual patients. Therefore, the adaptation of personalized medicine is vitally important to pave the way for effective treatment.

Overall, our topic Research Topic not only provided a comprehensive and in-depth discussion of current knowledge about the ALS-FTD spectrum, but also presented exciting new findings from several original research articles. We anticipate that this Research Topic will promote further investigation of disease mechanisms and shed light on exploring effective therapeutic approaches for this devastating disorder.

## Author Contributions

WG wrote the draft of the manuscript. Z-YZ, YL, D-DS, and SC contributed to manuscript revision, read, and approved the submitted version. All authors contributed to the article and approved the submitted version.

## Funding

WG holds a postdoctoral mandate fellowship from KU Leuven (PDM/18/213) and was supported by the Opening the Future Fund (KU Leuven), the Fund for Scientific Research Flanders (FWO, 1520019N), and the ALS Liga Belgium (A Cure for ALS). Z-YZ was supported by the National Natural Science Foundation of China (Grant No. 81974199). YL was supported by the National Institute on Aging (NIA) R00 AG056598. SC was supported by the Shanghai Shuguang Plan Project (18SG15), Shanghai Outstanding Young Scholars Project, and Shanghai Talent Development Project (2019044).

## Conflict of Interest

The authors declare that the research was conducted in the absence of any commercial or financial relationships that could be construed as a potential conflict of interest.

## Publisher's Note

All claims expressed in this article are solely those of the authors and do not necessarily represent those of their affiliated organizations, or those of the publisher, the editors and the reviewers. Any product that may be evaluated in this article, or claim that may be made by its manufacturer, is not guaranteed or endorsed by the publisher.
